# Autoimmunity: A New Focus on Nasal Polyps

**DOI:** 10.3390/ijms24098444

**Published:** 2023-05-08

**Authors:** Jingyu Huang, Yu Xu

**Affiliations:** 1 Department of Otolaryngology-Head and Neck Surgery, Renmin Hospital of Wuhan University, Wuhan 430060, China; 15129637209@163.com; 2 Research Institute of Otolaryngology-Head and Neck Surgery, Renmin Hospital of Wuhan University, Wuhan 430060, China

**Keywords:** autoimmunity, nasal polyps, chronic rhinosinusitis, autoantibodies, *Staphylococcus aureus*, superantigens

## Abstract

Chronic rhinosinusitis with nasal polyps (CRSwNP) has long been considered a benign, chronic inflammatory, and hyperplastic disease. Recent studies have shown that autoimmune-related mechanisms are involved in the pathology of nasal polyps. Activated plasma cells, eosinophils, basophils, innate type 2 lymphocytes, mast cells, and proinflammatory cytokine in polyp tissue indicate the mobilization of innate and adaptive immune pathways during polyp formation. The discovery of a series of autoantibodies further supports the autoimmune nature of nasal polyps. Local homeostasis dysregulation, infection, and chronic inflammation may trigger autoimmunity through several mechanisms, including autoantigens overproduction, microbial translocation, molecular mimicry, superantigens, activation or inhibition of receptors, bystander activation, dysregulation of Toll-Like Receptors (TLRs), epitope spreading, autoantigens complementarity. In this paper, we elaborated on the microbiome-mediated mechanism, abnormal host immunity, and genetic changes to update the role of autoimmunity in the pathogenesis of chronic rhinosinusitis with nasal polyps.

## 1. Introduction

Autoimmune diseases are a group of diseases that elicit specific immune responses against the self component. Approximately 3 to 5 percent of the population suffers from various autoimmune diseases [1,2]. Normally, the immune system can distinguish self from non-self, harmless non-self from dangerous non-self. However, genetic, infections and other environmental factors can cause loss of self-tolerance through mechanisms such as autoantigen overproduction, epitope spreading, and molecular mimicry. Consequently, the immune system induces an excessive immune response against the host’s normal cells and tissues, leading to inflammation and tissue damage [3], a phenomenon called autoimmunity.

Chronic rhinosinusitis (CRS) is an inflammatory disease that affects more than 10% of the population and is subdivided into chronic rhinosinusitis with nasal polyps (CRSwNP) and chronic rhinosinusitis without nasal polyps (CRSsNP), depending on the presence or absence of nasal polyps. Symptoms such as nasal congestion, nasal discharge, facial pain/pressure, and reduced sense of smell seriously impair the patient’s quality of life, with more than one million surgical interventions performed worldwide each year [4]. Recent research indicates that autoimmune mechanisms are involved in immune system disorders in CRSwNP [5,6]. We searched and filtered relevant literature related to “autoimmune” and “Chronic Rhinosinusitis with Nasal Polyps (CRSwNP)” using subject terms and free terms in the PubMed, Embase, and Cochrane Library databases, resulting in 83,251 and 4 relevant articles, respectively. Previous studies have shown that local self-reactive B cells and plasma cells increase in patients with CRSwNP compared to controls [7]. Furthermore, an increased level of anti-DNA double-stranded (anti-dsDNA) IgG in nasal polyp tissues is associated with IgE and eosinophilic cationic protein (ECP), representing more severe CRSwNP phenotypes, such as aspirin-exacerbated respiratory disease or aggressive CRSwNP [5,6,8,9]. Autoimmune-associated nasal polyps have appeared as part of Wegener’s Granulomatosis (WG), an autoimmune-associated systemic vasculitis associated with proteinase 3 anti-neutrophil cytoplasmic antibodies (PR3-ANCAs) [10,11]. However, the critical question remains: Is there sufficient human evidence to classify all or most types of nasal polyps as an autoimmune-related disease?

Some studies have shown a close relationship between CRSwNP and autoimmunity, but many questions remain unanswered. Therefore, this review aims to discuss the autoimmune mechanisms of nasal polyps from a descriptive point of view and summarize the autoimmune-related pathogenesis of CRSwNP.

## 2. Methods

In order to ensure a comprehensive and thorough review of the literature, the authors conducted a rigorous search of the PubMed, Embase, and Cochrane Library databases using a combination of mesh and non-mesh terms related to nasal polyps and autoimmunity. Specifically, the mesh terms “Nasal Polyps” [Mesh] and “Autoimmunity” [Mesh] were used, along with frequently used non-mesh terms such as “nasal polyp OR chronic rhinosinusitis with nasal polyp OR chronic rhinosinusitis and nasal polyposis OR nasal polyposis” and “autoimmune OR autoantibod* OR autoantigen*”. A total of 338 articles were initially retrieved, and their titles and abstracts were screened by two independent researchers to assess their relevance. Articles that addressed the relationship between nasal polyps and autoimmunity, as well as any specific autoimmune mechanisms, were further screened in their full-text version, and reference lists of retrieved publications were also reviewed.

This search strategy was also used in other chapters of the review to ensure consistency and completeness. To ensure a thorough search, the authors also used additional keywords related to specific autoimmune mechanisms in conjunction with nasal polyps, such as microbiome, *Staphylococcus aureus*, nasal vaccines, matrix metalloproteinases (MMP), neutrophil extracellular traps (NETs), molecular mimicry, inhibition receptors, bystander activation, Toll-like receptor (TLR), epitope spreading (ES), autoantigen complementarity, single nucleotide polymorphisms (SNPs), epigenetic modifications. Any disagreements on article inclusion were resolved by two independent researchers.

## 3. Relevance of Autoantibodies in the Autoimmune Damage in Nasal Polyp

The binding of autoantibodies to autoantigens interferes with the normal function of critical proteins or pathways and triggers classical antibody-mediated complement activation pathways to form membrane attack complexes, leading to cell death. Autoantibodies against the nucleus, basement membrane antigen, extracellular matrix (ECM), phospholipids, and cytokines have been found in nasal polyps [6,9,12,13]. Most of the increased autoantibodies are limited to nasal polyp tissue extracts, while there is no such phenomenon in the inferior turbinate of CRSwNP patients. Anti-BP180 autoantibodies were locally found in nasal polyps [9]. BP180 (BPA2/collagen XVII) is a transmembrane glycoprotein that maintains the adhesion of the layered epithelium to the basement membrane, and these autoantibodies may play a role in the loss of epithelial barrier function. In 138 asthma patients, 12(8.7%) patients were detected with anti-periplakin IgE antibodies, and the frequency of occurrence was greater in patients with nasal polyposis (*p* < 0.05) [14]. Periplakin (PPL) is a component of desmosomes that plays a role in maintaining epithelial cohesion, intracellular signal transduction, and antigen presentation. Eide et al. found that elevated levels of antiphospholipid (APA) antibodies in nasal polyps trigger the coagulation cascade that leads to extravascular fibrin deposition in nasal polyp tissue. This elevation is also seen in systemic lupus erythematosus (SLE), rheumatoid arthritis (RA), scleroderma, and systemic vasculitis [13]. The increase of IL-5 levels in CRSwNP patients is associated with chronic inflammation and edema in the nasal mucosa of patients with nasal polyps. Tsybikov et al. found that the increase in IL-5 in CRSwNP patients was associated with the induction of anti-IL-5 autoantibodies [12]. CRSwNP with T2 endotype is chronic mucosal eosinophil inflammation with Th2 bias, suggesting cytokine autoantibodies may influence Th1/2 imbalance [12]. The specific antibodies detected in CRSwNP and their roles in the occurrence of nasal polyps are shown in Table 1. Antibody-mediated cascade activation of complement is increased in nasal polyp tissues of CRSwNP patients [8]. Complement activation can induce cell lysis of non-nucleated cells (such as bacteria and red blood cells), activate nucleated cells and/or promote tissue damage [15], and promote epithelial-mesenchymal transformation (EMT) to cause nasal mucosal tissue remodeling and accelerate nasal polyps formation [8]. The evidence above supports the critical role of local activation and antibody production of B cells in the pathogenesis of CRSwNP.

## 4. CRS and Human Microbiome

### 4.1. Changes in the Microbiota of CRS

Autoimmune responses can be promoted or blocked by symbiotic microorganisms [18]. Studies have also shown that microbiome-induced autoimmune reactions are associated with disorders that are not normally considered autoimmune such as inflammatory bowel disease (IBD), asthma, autism, depression, and schizophrenia [19]. In CRS patients, the diversity, richness, and evenness of bacterial community are significantly reduced compared to healthy controls [20,21], and these patients usually present an expansion of pathogenic bacteria and a reduction of symbiotic bacterial populations [22]. Furthermore, several studies have shown that CRS patients have higher rates of *Staphylococcus aureus*, *Corynebacterium* spp., and various anaerobic species compared with healthy controls (see details in Table 2) [20,23,24,25], leading to the destruction of the nasal epithelial barriers and excessive immune responses. Dysbiosis, inflammation, and overactivated immune responses interact locally in the nasal cavity and may lead to systemic inflammatory processes (including autoimmune manifestations). *Staphylococcus aureus* (the key pathogen of CRS) can be influenced by communication with normal symbiotic microorganisms [26]. Some studies found that the growth of *Staphylococcus aureus* in CRS can be inhibited or promoted by actinomycetes (such as *Corynebacterium*) [27], *Proteus* spp. (mainly *Escherichia coli*), *Staphylococcus epidermidis* [28], and *Propionibacterium acnes* [26]. *Staphylococcus aureus* is more common in the nasal mucosa and nasal polyp tissue of CRSwNP than in CRSsNP [20,29]. Mucosal immune deficiency, colonization of *Staphylococcus aureus*, and the formation of complex microbial flora results in the sustained mucosal inflammatory response [30].

### 4.2. Impact of Nasal Vaccines on Nasal Microbiota and CRS

In recent years, nasal vaccines have become increasingly popular as potential alternatives to traditional injectable vaccines. These vaccines are administered through the nasal cavities and have higher compliance rates, lower risk of infection, lower cost, and easier large-scale use than traditional injectable vaccines [34]. Nasal vaccines have been found to be effective in treating Alzheimer’s disease and other neurodegenerative diseases, cancer, and autoimmune diseases [34,35,36]. Moreover, several studies have shown that nasal vaccines can significantly impact the nasal microbiota by changing its abundance and diversity, which may have an impact on health [37,38]. While some changes may improve the immune system’s ability to cope with infection, other changes may increase the risk of secondary infections or other health problems. Nasal bacteria play an important role in inducing virus-specific adaptive immunity [39]. Some studies show that mucosal influenza-specific IgA is significantly correlated with some bacteria, such as *Streptococcus pneumoniae*, *Bifidobacterium animalis*, and *Lactobacillus casei*, which have been studied as mucosal adjuvants for influenza vaccines in mice [40]. The use of such adjuvants not only strengthens systemic and mucosal protective immune responses but also extends protection against different influenza subtypes [41,42]. Therefore, the interaction between nasal vaccines and nasal microbiota is complex. The effectiveness of nasal vaccines in the prevention and induction of diseases such as CRSwNP remains uncertain. Patients with CRS often have pneumococcal antibody deficiency, which can be corrected by vaccination with PPSV23 [43]. Studies have shown that after vaccination with PPSV23, the incidence of chronic rhinosinusitis and recurrent acute rhinosinusitis decreased, and prescriptions for antibiotics and corticosteroid drugs decreased by about 20% [43,44]. Nasal vaccines can affect the balance of natural microbiota in the nasal cavity, but whether disruption of microbiota balance by nasal vaccines promotes the occurrence of CRSwNP has not been directly proven. But healthcare providers should monitor patients receiving nasal vaccines for any signs of nasal polyps or other adverse reactions.

### 4.3. Microbial-Dependent Mechanisms of Autoimmunity

#### 4.3.1. Autoantigens Overproduction

**Figure 1:**
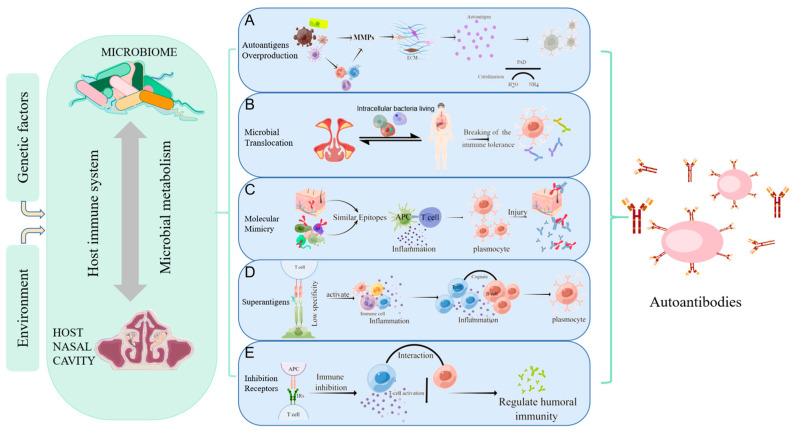
Mechanisms of autoimmunity triggered by microorganisms. Host-microbe interaction can be influenced by environmental and genetic factors, causing abnormal immune regulation, and affecting the local or systemic autoimmune regulation. (**A**) Autoantigens Overproduction: MMPs secreted directly by bacteria or by immune cells stimulated by bacteria can degrade the extracellular matrix to produce “remnant epitopes” acting as autoantigens. Or the protein fragments produced by MMPs can be modified (such as citrullination) to form antigen epitopes; (**B**) Microbial Translocation: bacteria and their products can migrate to other organs using some substitute for dissemination mechanisms(such as intracellular bacteria living), resulting in abnormal contact with the host immune system, local inflammatory responses, and the production of autoantibodies; (**C**) Molecular Mimicry: molecular mimicry occurs when self-antigen has the similar epitopes in composition or structure with microbial epitopes. When the immune system attacks microorganisms, it accidentally injures host cells due to the similarity of antigens; (**D**) Superantigens: superantigens such as staphylococcus aureus enterotoxin b (SEB) interact with the Vβ variable region of the T cell receptor (TCR), leading to the expansion of T cell. This less specific recognition can mediate a powerful inflammatory response and induce normal resting autoreactive T and B cells activate to induce the autoimmune response; (**E**) Inhibition Receptors Related to Autoimmunity: inhibitory receptors regulate autoimmunity by inhibiting the immune response and limiting TCR signaling. Nasal mucosa T cells are not controlled after inhibitory receptors (PD-L1, PD-L2) are reduced, producing IL-21 to promote B cell activation and excessive IgM, IgA, and IgE production, as well as granulocyte inflammation. (Figdraw was used to create the Figure 1).

The overproduction of autoantigens is presumably due to the enzymes produced by microorganisms that decompose the extracellular stroma (fibronectin, fibrinogen, type I collagen), producing “remnant epitopes” recognized as autoantigens by major histocompatibility complex class I (Figure 1A). Alternatively, it can be processed and presented to autoreactive T cells and stimulate B cells to produce autoantibodies [45]. For example, *P. gingivalis* culture supernatants effectively decompose extracellular stroma in arthritic joints and oral mucosae, giving rise to neoepitopes to drive the production of autoantibodies [46]. Microbial infection and local chronic inflammation activate circulating immune cells to produce matrix metalloproteinases (MMPs), causing the degradation of matrix molecules, which is an integral part of generating residual epitopes [47]. When analyzing the biological function of intracellular MMP-9 substrates, two-thirds of candidates were autoantigens in cancer or autoimmune diseases [48]. In multiple sclerosis, α B-Crys are myelin components that cause autoimmune responses, and MMP-9 cleaves α B-Crys at multiple sites in vitro and releases immunodominant and recessive epitopes of α B-Crys [48].

The upregulation of MMPs is closely related to tissue remodeling and polyp formation in allergic airways [49]. Previous studies have demonstrated increased expression of MMP-1, -2, -3, -7, -8, and -9 in the nasal polyp tissues of patients with CRSwNP, and upregulation of MMP-9 expression in CRSwNP is widely reported [49,50,51,52,53]. Additionally, infection with *Staphylococcus aureus* also leads to upregulation of MMP-1 and MMP-9 [51]. When local homeostasis is out of balance, these high levels of proteases lead to the infiltration of inflammatory cells and interstitial edema in CRSwNP. MMPs also can induce epitope production when modulating matrix composition. Neutrophil extracellular traps (NETs) are significantly elevated in nasal polyps, playing a key role in neutrophil inflammation in CRSwNP [54]. NET-associated proteases, neutrophil elastase, and MMP-9 remodel laminin epitopes, activate integrin alpha-3beta-1 signaling, and induce dormant cancer cell proliferation and metastasis [55]. Previous studies have found that MMP12-generated elastin fragments act as an autoantigen to drive the autoimmune process in chronic obstructive pulmonary disease (COPD) mouse models [56]. These findings provide experimental evidence of MMP-mediated autoimmunity in the upper airway. Therefore, enzymes produced by microbiota and MMPs produced by host immune cells can create residual epitopes, acting as autoantigens or pathway activators, which may play a vital role in the pathogenesis of nasal polyps.

On the other hand, MMP-generated protein fragments are modified to form antigen epitopes, such as citrullination. Citrullination can induce the production of anti-citrullinated protein antibodies (ACPA) and drive the autoimmune response [57,58]. Since the ACPA also cross-react with peptides that undergo other post-transcriptional modifications, such as carbonylation and acetylation, the range of epitopes recognized by ACPA is further extended [57,59]. Cigarette smoke accelerates the progression of arthritis in collagen-induced arthritis (CIA) models, while anti-cyclic citrullinated peptide (anti-CCP) antibodies increase in the lung tissue, hock joint, and serum in the smoke-aggravated CIA mice [60]. In ovalbumin (OVA)-immunized asthmatics/allergic mice, 2CA (peptidylarginine deiminase [PAD] inhibitor, PAD enzyme is used to catalyze citrullination) significantly inhibited lung tissue protein citrullination, inflammatory cell recruitment, and airway Th2 cytokine secretion, and 2CA also significantly inhibited systemic OVA-specific IgE and total IgE production [61]. These studies illustrate that the excessive production of antigen epitopes can aggravate the inflammatory manifestations in airway diseases. Reducing antigen modification and epitope production may alleviate airway inflammatory diseases.

#### 4.3.2. Microbial Translocation

Many bacteria can use some substitute for dissemination mechanism, including intracellular bacteria living within host cells (*Listeria monocytogenes*, *Salmonella typhimurium*) [62] and the use of circulating neutrophils as Trojan horses (*Staphylococcus aureus*) [63], leading to abnormal contact with the host’s immune system (Figure 1B). This translocation process, called microbial translocation, can cause inflammation and tissue damage, and trigger autoimmunity. *Staphylococcus aureus* has been confirmed to survive in macrophages, endothelial cells, neutrophils, and even some strains of *S. aureus* can survive and replicate in the host cells, then lysing the target cells and appearing in other organs [63,64].

Antibodies against *Helicobacter pylori,* which is known to colonize the human stomach, react with extra-gastric tissues, such as glomerular capillary walls, renal tubular cells, and glomerular basement membranes, from patients with lupus nephritis [65]. Furthermore, a study found that the translocation of *Staphylococcus* can promote B cell activation and induce autoantibodies production by enhancing germinal center response in patients and animals with human immunodeficiency virus (HIV) [66], suggesting that microorganisms and their components can translocate to local tissues and participate in the development of autoimmunity.

A study in patients with CRS and cystic fibrosis found reduced diversity of sinus flora and a loss of niche specificity between the upper and lower airways, supporting the concept of microbial translocation for airway disease [67]. Continuous delivery of staphylococcal enterotoxin B by subcutaneous microcosmic pumps in HLA-DQ8 transgenic mice resulted in a multi-system autoimmune inflammation characterized by mononuclear infiltration of the lungs, livers, and kidneys, accompanied by antinuclear antibodies production and immune complexes deposition in the glomerulus, similar to systemic lupus erythematosus [68]. This type of staphylococcal enterotoxin B exposure occurs naturally in *Staphylococcus aureus* (a key pathogen that causes CRSwNP) carriers. Therefore, the interrelationship between the translocation of nasal microorganisms, autoimmunity, and nasal inflammatory diseases should be explored.

#### 4.3.3. Molecular Mimicry

Molecular mimicry occurs when the hosts and microbes share similar epitopes (Figure 1C) [69]. The homology of many microbial peptides/proteins with human tissue peptides/proteins has been extensively studied, and up to 99.7% of viral and bacterial peptides overlap with the human proteome [70]. For example, patients with Guillain-Barre syndrome infected with *Campylobacter jejuni* can generate antibodies against ganglioside complexes through molecular mimicry [71]. Antibodies against complementary Pr3 (cPr3) have been found in anti-neutrophil cytoplasmic antibodies(ANCA)-associated vasculitides, and some bacterial proteins are homologous to cPr3 polypeptides, including two proteins found in Staphylococcus [72]. WG intranasal lesions (such as nasal polyp) are associated with a higher colonization rate of intranasal *Staphylococcus aureus*, which promotes mucosal barrier defects, the formation of chronic inflammation and granuloma in the upper respiratory tract [73].

Heat shock proteins (HSP) are highly conserved proteins with strong immunogenicity that are induced during stress. The online T-coffee multiple sequence alignment tools (EMBL-EBI) of *Mycobacterium aviumsubsp paratuberculosis* (MAP) HSP65 and human HSP60 indicated 97% identity [74]. Therefore, Hsp65 released by *Mycobacterium aviumsubsp paratuberculosis* can induce T cells to attack autoantigens, leading to autoimmune diseases such as atherosclerosis, adjuvant arthritis, diabetes, and so on [75]. HSP70 autoantibodies and HSP60 autoantibodies are present in asthma patients’ plasma, and the presence of HSP70 autoantibodies is associated with the onset and severity of asthma [76]. In contrast to non-allergic rhinitis (AR) patients, HSP70 increases in nasal lavage fluid of AR patients [77]. Many autoantigens, such as human manganese superoxide dismutase (MnSOD), ribosomal P2 protein, cyclophilin (CyP), and thioredoxin, show structural similarities with environmental allergens and have significant positive results in skin tests [78]. One study found that IgE from patients with allergic bronchopulmonary aspergillosis (ABPA) can recognize human thioredoxin as an autoantigen, suggesting amino acids shared between human and fungal thioredoxin are involved in IgE binding and confirming that IgE-mediated cross-reactivity and autoreactivity are involved in chronic atopic disease by molecular mimicry [79]. Previous studies have found increased HSP70 and anti-HSP70 sIgA in nasal secretions of CRSwNP, and the concentration is associated with the Lund-Mackay score in the CRSwNP group but not in the CRSsNP group [17]. Molecular mimicry may be one of the autoimmune mechanisms of CRSwNP. However, strong evidence is needed to prove the role of symbiotic bacteria as shared antigen providers in these cross-reactions of molecular mimicry in nasal polyp disease models.

#### 4.3.4. Superantigens

Microbial superantigens (SAg) are microbial proteins that directly bind to the V region of TCR β-chains with MHC class II molecules (MHC-II) [80]. Superantigen (SAg) differs from conventional antigens by binding outside the complementary determining regions (CDRs) to induce cell activation through receptor cross-linking. Compared with conventional antigen interactions, the β-chain CDR2 loop does not undergo V(D)J recombination in activating T cells in a TCR Vβ-specific manner (Figure 1D) [81]. The specificity of T cells in SAg recognition is significantly reduced. Thus, SAg can activate CD4^+^ and CD8^+^ T cells, iNKT cells, MAIT cells, γδ T cells, etc. [82,83,84]. They can also induce autoimmunity by activating antigen-presenting cells (APCs) and normal resting autoreactive T and B cells, produce a large number of regulatory and effector cytokines, and change the immune function of the host [68].

Staphylococcus aureus enterotoxins (SEs) and Toxic Shock syndrome Toxin-1 (TSST-1) in *Staphylococcus aureus* are commonly described as superantigens [63]. Recent studies have shown that SEs and TSST-1 may activate a large number of CD4^+^ cells and are associated with various autoimmune diseases such as multiple sclerosis (MS), WG, and RA [63,85]. In patients with acute infection of TSST-1-producing *S. aureus*, Vβ2-TCR^+^ T cells may expand from 10% to 70% of all T cells [86], initiating an immune response via a “bridge” between MHC II and TCR-Vβ regions [87], thus bypassing normal antigen processing and presentation mechanisms. In addition, self-reactive B cells will be activated by staphylococcal enterotoxin B (SEB) and release cytokines, leading to homologous T, B cells interactions and B cells activation [85], while T cells do not need to recognize specific autoantigens to assist B cells. SEB plays a crucial role in activating specific autoreactive Vβ8^+^ T cells and producing TH1 cytokines, which may be an important mediator of rheumatoid inflammation and joint destruction [88].

SEB, as a superantigen, is known to significantly increase the recruitment of nasal mast cells in the nasal microenvironment [86]. The activation products of these mast cells, including prostaglandin D2 (PGD2) and others, have been found to cause inflammation and damage to the nasal epithelium. Internalization of *S. aureus* in mast cells causes a significant increase of *S. aureus* in the subepithelial layer and promotes a chronic inflammatory environment [89]. Gevaert et al. confirmed the presence of secondary lymphoid tissue, IgE antibodies to S. aureus enterotoxin (SAE), and polyclonal hypersensitive immunoglobulin E in polyp tissue, which were related to *Staphylococcus aureus* colonization and tissue eosinophilia [90]. The formation of secondary lymphoid tissues promotes B cell activation and facilitates local antibody production to induce autoimmunity [91]. All these phenomena indicate that *S. aureus* superantigen-mediated autoimmunity plays a proinflammatory role in CRSwNP. Min-Seok Rha et al. found that Staphylococcus aureus enterotoxin I could be a major superantigen to drive the expansion of TCRVβ5.1^+^ CD4^+^ T cells in CRSwNP patients [92]. These T cells exhibited significant proliferative characteristics and TH2 phenotypes, and the proportion of TCRVβ5.1^+^ or Vβ1^+^CD4^+^ T cells was correlated with Lund-Mackay CT scores, predicting more severe nasal polyps [92]. Additionally, nasal colonization of *S. aureus* has been associated with specific phenotypes of SLE, such as high frequency of different autoantibodies (anti-dsDNA, anti-Sm, anti-SSA, anti-SSB, and anti-RNP) and extensive renal and skin involvement [93]. Although autoimmune reactions have been confirmed in nasal polyps [6], the specific mechanism of superantigen’s effects on autoimmunity in nasal polyps still needs to be further study. By evaluating the crucial role of superantigens in the regulation of immune cells, the mechanisms of microorganisms in nasal polyps and other autoimmune diseases can be revealed.

#### 4.3.5. Inhibition Receptors Related to Autoimmunity

In healthy mice, up to 30% of CD4^+^FOXP3^−^ cells can respond to self-antigens but are inhibited by Tregs [94]. In mice lacking Tregs, compensatory expression of inhibitory receptors (IRs) has been proposed [94]. The role of IRs is to limit TCR signaling, inhibit immune responses, and limit the recognition of autoantigens after infection (Figure 1E). Currently, well-studied IRs include cytotoxic T-lymphocyte-associated protein 4 (CTLA4), Programmed cell death protein 1 (PD-1), and Lymphocyte Activating Gene 3 (LAG3) [95]. In non-autoimmune predisposition models, gene ablation of IRs (CTLA4, PD1, LAG3) causes spontaneous autoimmune symptoms, albeit with slightly different manifestations [96,97,98]. Single nucleotide polymorphisms (SNPs) in CTLA4 are strongly associated with autoimmune diseases such as Type 1 diabetes mellitus (T1D), Graves’ disease, and lupus erythematosus (SLE) [99].

The function of IRs is influenced by the microbiota in vivo. Studies have shown that the anticancer effect of CTLA-4 blockade depends on different *Bacteroides* species, as sterile tumor mice or those treated with antibiotics did not respond to CTLA blockade [100]. Lukas et al. suggested that specific bacteria could use the immune system to kill cancer cells by regulating inosine production and mediating IRs [101]. CTLA-4-related antigens have been shown to directly inhibit *Staphylococcus aureus*-induced B cell activation and interfere with T-B cell interaction, thereby regulating humoral response in vitro [102].

Compared with peripheral blood mononuclear cells (PBMC), the expression of CTLA-4^+^ Treg cells in local lymphocytes of nasal polyps was significantly increased [103]. Single-cell analysis of nasal polyps from patients with CRSwNP found that inhibitory receptors, including LAG3 and CTLA4, were significantly overexpressed in the TH2 TCN1 cells [104]. PD-1, a negative T cell regulator, maintains peripheral tolerance and transmits negative signals to T cells when interacting with ligands [105]. PD-1 expression is higher in nasal polyps than in the control group, particularly in patients with asthma, and the expression level correlates with the severity of the disease [106]. The accumulation of PD-1^hi^CXCR5^−^CD4^+^ T cells outside ectopic lymphoid tissues has been found in nasal polyp tissue, but not in the peripheral blood in patients with CRSwNP [107]. Reduction of PD-L1 and PD-L2 levels promotes the expansion of PD-1^hi^ CXCR5^−^CD4^+^ T cells [108]. PD-1^hi^CXCR5^−^CD4^+^ T cells are not located in ectopic lymphoid tissue, providing a mechanism for local immunoglobulin production in 80% of nasal polyps without ectopic lymphoid tissue [107]. When PD-L1 or PD-L2 are reduced, nasal mucosal T cells are uncontrolled and produce pathogenic cytokine IL-21 that promotes B cell activation and excess IgM, IgA, and IgE production, as well as granulocytic inflammation [107]. It is puzzling why the PD-1^hi^CXCR5^−^CD4^+^ T cells are not kept in check by the high expression of PD-1, yet are highly activated in polyp tissues. This could be due to the lack of notable expression of Treg cell markers in PD-1^hi^CXCR5^−^CD4^+^ T cells, such as Foxp3 and CD25 [107]. Wang et.al identified PD-1hiCXCR5^−^CD4+ T cells in nasal polyps that can be characterized by expression of IFN-γ, IL-17A, and IL-4, and PD-1^hi^CXCR5^−^CD4^+^ T cells can induce IgE production via IL-4 only in eosinophilic NPs [107]. Circulating PD-1^hi^CXCR5^−^CD4^+^T cells are associated with autoimmune diseases, such as arthritis and systemic lupus erythematosus, and are positively correlated with disease activity in both cases [105,109]. However, the expansion of PD-1^hi^CXCR5^−^CD4^+^ T cells in tumor immunity has been associated with increased tumor-infiltrating lymphocytes (TIL) and long-term survival [109]. These findings prompt us to consider whether nasal polyps may be a localized autoimmune disease with a Th2 phenotype.

## 5. Mechanism of Autoimmunity Dependent on the Host Response

### 5.1. Bystander Activation

**Figure 2:**
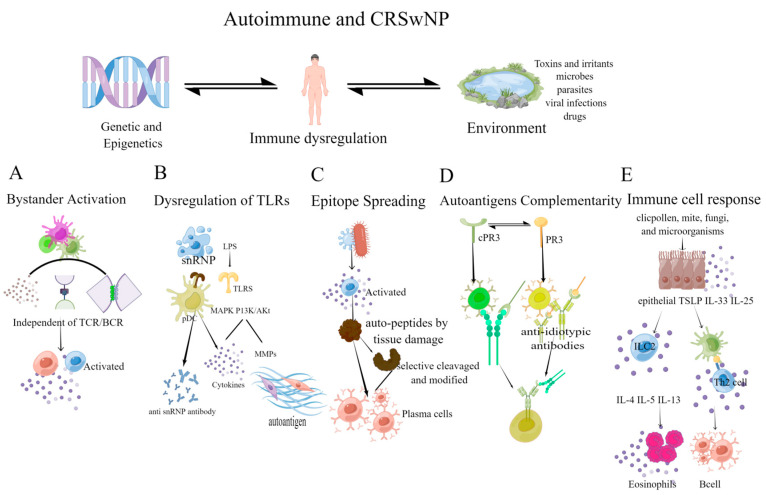
The mechanism of autoimmunity depends on the host response, resulting in the loss of immunological tolerance to self-antigens. (**A**) Bystander activation: this activation is independent of TCR and BCR. The inflammatory microenvironment activates bystander immune cells, perpetuating the inflammatory environment and forming a vicious cycle; (**B**) Dysregulation of TLRs: snRNPs can stimulate TLRs, activate innate immune cells, such as plasmacytoid dendritic cells (pDCs), and promote the production of anti-SnRNPs autoantibodies [104]. TLRs can affect local inflammation, and promote the production of MMPs through MAPK and PI3K/Akt signaling pathway, leading to the emergence of autoantigen epitopes; (**C**) Epitope Spreading: inflammation, tissue damage, and the release of autopeptides can activate T cells to recognize epitopes and activate B cell complementarity, causing epitope spreading. The selective cleavage or epitope modification by proteases in APCs also contributes to epitope spreading; (**D**) Autoantigens Complementarity: the autoantigen has a complementary amino acid sequence to the initial antigen sequence. The initial protein initiates the production of antibodies and causes an anti-antibody response (the anti-idiotypic response), resulting in anti-idiotypic antibodies reacting with the autoantigen; (**E**) Immune cell response: mites, fungi, microorganisms, etc., induce epithelial cells to secrete cytokines that activate innate lymphoid type-2 cells (ILC2) and TH2 cells, causing local eosinophilia inflammation and B cell activation. (Figdraw was used to create the Figure 2).

Bystander activation refers to a heterogenic activation of immune cells that is independent of antigen and TCR/BCR specificity. This type of activation is mediated by indirect signals from the inflammatory environment, including co-signaling receptors ligands, cytokines, chemokines, pathogen-associated molecular patterns, and extracellular vesicles with microbial particles (Figure 2A) [110]. Bystander activation has been linked to the development or recurrence of various autoimmune diseases, such as rheumatoid arthritis [111], lupus erythematosus [112], multiple sclerosis [113], and autoimmune thyroid diseases [114]. Cytokines produced in the inflammatory microenvironment activate bystander T cells to secrete IL-2, IL-6, and TNF-α, perpetuating the inflammatory environment and forming a vicious cycle [115]. This cycle plays a crucial role in maintaining the synovial inflammatory cycle in RA patients [115]. The increased expression of TRIM-21 in B cell lines stimulated with Der p 2 may further enhance the response of B cell lines, leading to bystander activation of the inflammatory process in house dust mite (HDM) allergic SLE patients [112]. These experimental findings can provide valuable insights for studying autoimmunity in otorhinolaryngology. The translocation of *Staphylococcus aureus* can promote B cell activation and induces autoantibodies production by enhancing germinal center response. These autoantibodies are not produced by antigen-specific B cells but by activated bystander B cells [66]. Infection with *Staphylococcus aureus* can prevent the successful acceptance of skin allografts by inducing inflammation and IL-6 production, triggering the bystander activation of alloreactive T cells, and precipitating acute allograft rejection [116]. Bystander activation provides a theoretical basis for targeting *Staphylococcus* to prevent autoantibodies production in the nasal polyp disease model. Bystander activation mechanisms also involve other factors such as microbial translocation and superantigen, which may trigger local cytokine storms and cause non-specific activation of immune cells.

### 5.2. Dysregulation of TLRs

Toll-like receptors (TLRs) are transmembrane protein receptors belonging to the pattern recognition receptor (PPR) family, which can recognize a wide range of pathogen-associated molecular patterns (PAMPs). TLR can induce the production of pro-inflammatory cytokines, chemokines, and the expression of costimulatory molecules to prevent microbial invasion [117]. Many studies have demonstrated the crucial role of TLRs in various autoimmune diseases. Human plasmacytoid dendritic cells (pDCs) stimulated by sera autoantibodies to small nuclear ribonucleoprotein particles (snRNPs) can produce IFN via endosomal TLRs (TLR7, TLR8, and TLR9) to aggravate local damage in SLE and psoriasis [118,119]. Compared with healthy controls, several TLRs, including TLR2, TLR3, TLR5, TLR6, TLR7, and TLR9, are expressed in the synovium of RA patients, which can induce the production of multiple inflammatory chemokines [120,121,122]. The occurrence of single nucleotide polymorphisms in DNA sequences encoding TLRs can lead to the malfunction of some key signaling pathways, as a result, increases the risk of autoimmune diseases. For instance, both type 1 diabetes and allergic asthma have been significantly associated with a polymorphism rs3804100 (S450S, +1350) in TLR2, which may share a common susceptibility locus [123,124].

Activation of TLRs in human nasal epithelial cells (HNECs) promotes the production of cytokines, including thymic stromal lymphopoietin (TSLP), IL-33, and IL-25. These cytokines can activate type 2 immune responses by inducing the proliferation of innate lymphoid type-2 cells (ILC2) and TH2 cells [125]. There have been many studies on TLRs in nasal polyps, but there is no consistent conclusion on the expression levels of TLRs in CRSsNP and CRSwNP. Most studies indicate that compared with CRSsNP patients or control subjects, patients with CRSwNP have significantly increased TLR2, TLR4, and TLR7 [126,127]. Cho et al. found that LPS triggers the immune response through TLR4, activating MAPK and PI3K/Akt signaling pathway and promoting the production and expression of IL-6, IL-8, and MMP-1 in nasal polypus-derived fibroblasts and nasal polyp tissues (Figure 2B) [128]. Activation of TLR4 promotes the production of vascular endothelial-derived growth factor (VEGF) [129] and contributes to the remodeling of nasal polyps, while TLR3 activation induces the production of type I interferon and TSLP, exacerbating the type 2 immune response and promoting airway epithelial inflammation [130,131]. The local inflammatory environment and production of MMPs may lead to the excessive production of local autoantigens, thereby promoting autoimmunity [56]. Collectively, these findings suggest that TLRs and their regulatory mechanisms are involved in developing nasal polyps.

### 5.3. Epitope Spreading

Epitope Spreading (ES) refers to the diversification process of immune response from primary dominant epitope to secondary epitope over time, thereby improving the efficiency of the host immune system in protecting against non-self antigens [132]. However, immune responses induced by major epitopes on antigens, such as viruses, may extend to include epitopes on autoantigens during inflammation. In genetically predisposed individuals, epitope spreading may trigger an autoimmune response. There are two main mechanisms underlying the occurrence of ES in B cells: in the “independent” mechanism, persistent infection can cause activation of microbial-specific T cells, independent of APCs, which mediates tissue damage and the release of auto-peptides. Inflammation and auto-peptides can induce T cells to recognize covert epitopes and activate B cells, leading to epitope spreading. The “dependence” mechanism activates T cells and B cells by processing and presenting associated antigens, with APCs contributing to epitope spreading through selective cleavage molecules by proteases in lysosomes or other mechanisms (Figure 2C) [132,133].

Potential autoantigens are antigen targets after modification by reactive oxygen species [134], lipid oxidation [135], abnormal cell apoptosis [136], and citrullination. Oxidatively modified autoantigens are caused by an imbalance in the pro-oxidant/antioxidant, resulting in an increase of oxidative stress and the release of peroxidation products. Aldehydes, mainly 4-hydroxy-2-alkenals, produced by oxidative modification, form adduct with proteins and are highly immunogenic [137]. Previous studies have found that oxidized autoantigens exist in various autoimmune diseases [138,139,140]. For instance, protein oxidative damage shows increased 4-hydroxy-2-nonenaldehyde modification in SLE patients [138]. A rapid autoimmune response and development of lupus-like disease were found in rabbits modified with 4-hydroxy-2-nonenal (HNE) modified 60 kD Ro autoantigen [134]. Peroxynitrite, a potent oxidant, and nitrating agent, inhibits the activity of tissue inhibitors of metalloproteinases, increases the activity of MMP-1 and MMP-3 [134], and promotes the overproduction of autoantigens. Oxidatively modified autoantigens, as new antigens, promote the loss of autoimmune tolerance and accelerate epitope spreading. Epitope spreading has not been extensively studied in the nasal cavity, but staphylococcus aureus enterotoxin A and oxidative stress play an important role in nasal polyps. Studies have shown that oxidative stress in serum and polyp tissues of patients with CRSwNP was higher than that of patients with CRSsNP [141,142]. ROS can activate TGF-β1 and contribute to the fibrogenic effects of TGF-β1, induce α-SMA expression, and collagen production [143]. ECM accumulation is a characteristic structural change observed in nasal polyps. Moreover, TGF-β1 can stimulate reactive oxygen species (ROS) production in various cells [144]. Oxidative stress has been linked to the secretion of inflammatory factors [145,146], and the inflammatory response of tissues leads to increased vascular wall permeability and extravasation of plasma proteins, ultimately causing edema and worsening nasal symptoms.

### 5.4. Autoantigens Complementarity

Autoantigen complementarity theory states that the immunogens that activate and lead to autoimmune disease are not the autoantigen or its mimics, but rather complementary protein–peptide. This complementary protein initiates the production of antibodies and induces an anti-antibody response, known as the anti-idiotypic response. The anti-diatypic antibody then reacts with autoantigen with an amino acid sequence complementary to the initial antigen sequence [147]. Idiotypic pairs have been identified in both mice and humans with anti-neutrophil cytoplasmic antibodies (ANCA) vasculitis. It has also been discovered that anti-idiotypic antibodies in these individuals bind to and interfere with plasminogen, increasing the risk of thrombosis (Figure 2D) [148]. Mice immunized with the human complementary PR3 peptide produce antibodies to the peptide’s sense counterpart, PR3 [147,148]. Furthermore, human and murine antibodies and their corresponding idiotypic antibody pairs can bind to each other, indicating that they can form anti-idiotypic pairs [149,150].

ANCA is a group of autoantibodies, primarily IgGs, involved in the pathogenesis of various autoimmune diseases. Among them, protease 3 (PR3) is the primary autoantigen of granulomatous polyvasculitis in the ANCA-associated vasculitis (AAV) family [148,151]. Around 60–70% of patients with PR3-granulomatosis with polyangiitis (GPA) are chronic nasal carriers of *Staphylococcus aureus* [152]. Staphylococcal proteins show homology to the cPr3 peptide [72]. Infection with these microorganisms can trigger or enhance immune responses to antisense PR3 peptide mimics and play an essential role in the pathogenesis of granulomatous polyvasculitis [153]. When pathogenic ANCA enters the circulation, they activate neutrophils by binding with ANCA antigens, activating neutrophils to produce reactive oxygen species (ROS), releasing proteolytic enzymes, and formating neutrophils extracellular trap (NET) [151] to release inflammatory factors such as IL-1β, TNF-α, platelet-activating factor, thromboxane E2, and leukotriene, causing tissue damage and immune overactivation [154]. While little research has been conducted on the association between nasal polyps and ANCA, most ANCA-positive CRSwNP patients are classified as eosinophilic granuloma with polyvasculitis (EGPA). Adult-onset of asthma, CRS, and tissue eosinophil infiltration in EGPA typically occur before the onset of systemic disease, and over 70% of patients have CRS with diffuse and bilateral nasal polyps [155]. These polyps share many similarities with eosinophilic chronic rhinosinusitis with nasal polyps (ECRSwNP) and are characterized by severe eosinophil infiltration, resulting in chronic recurrence even after surgery and drug therapy [156,157].

In conclusion, we can preliminarily conclude that autoantigens complementarity is the basis of ANCA production, leading to inflammatory responses that lead to the progression of the disease through bystander activation. However, as nasal polyps present as local manifestations in granulomatous polyvasculitis, further long-term prospective controlled studies and mechanistic studies are needed to explore the autoimmune mechanisms of nasal polyps.

### 5.5. Immune Cell Response

Type 2 immune responses, in which TH2 cells play a critical role, are critical in the pathogenesis of CRSwNP, especially in T2 CRSwNP. The production of type 2 cytokines such as IL-4, IL-5, and IL-13 promotes the synthesis of IgE by B cells and promotes the recruitment of eosinophils to the inflammatory site, leading to tissue damage and remodeling. The levels of autoantibodies in local nasal polyps are correlated with eosinophil levels and disease recurrence, suggesting that elevated autoreactive antibodies were associated with T2 CRSwNP [158]. Further studies are needed to explore the mechanisms by which autoreactive antibodies contribute to T2 CRSwNP pathogenesis. Th17 cells have been implicated in the development of autoimmune diseases and inflammation, while Treg cells inhibit these phenomena and maintain immune homeostasis [159]. Studies have shown that Th17/Treg ratio increases in patients with rheumatoid arthritis (RA), psoriasis, multiple sclerosis (MS), and inflammatory bowel disease (IBD) [160]. Correspondingly, non-T2 CRSwNP shows elevated levels of IFN-γ and IL-17 and an accumulation of TH1 and TH17 cells [161,162]. Treg cells regulate the activation of autoreactive T cells by downregulating the expression of CD80/86 on antigen-presenting cells (APCs) through CTLA-4 [163]. Treg deficiency can lead to the expansion of autoreactive T cells in nasal polyp tissue. Monoclonal antibodies targeting the Treg/Th17 imbalance have been used in clinical trials for other autoimmune diseases, such as psoriasis and rheumatoid arthritis, but have not been tested in CRSwNP [164]. SEB causes a significant increase of IFN-γ, IL-6, and IL-4, affecting the differentiation and function of Tregs [165]. Treg dysfunction plays an essential role in the occurrence and deterioration of CRSwNP. Treg dysfunction can lead to the imbalance between Th1/Th2 and Treg/Th17 in patients with CRSwNP, causing an imbalance between matrix metalloproteinases and their inhibitors. This imbalance can lead to the deposition of albumin, collagen, and other extracellular matrix proteins, and also can lead to autoantigens overproduction to mediate autoimmunity [166,167,168].

Activated B cells and plasma cells have been detected locally in the nasal cavity of CRSwNP patients, and tertiary lymphoid organs (TLOs) have been demonstrated locally in nasal polyps (Figure 2E) [91]. TLOs can activate autoreactive B cells and T cell clones, leading to chronic inflammation or the development of autoimmune. In addition, compared with tonsil B cells, nasal polyp-derived B cells secrete IgG, IgA, and IgE more frequently and abundantly [169]. In aspirin-exacerbated respiratory disease (AERD), an individual with the highest local IgE levels experiences the most rapid nasal polyp recurrence [170]. Tissue IgE can contribute to mast cell and basophil activation in the nasal cavity of patients with AERD [170]. Local overactivated B cell responses can activate complements, and this overactivated antibody-mediated complement activation is usually a hallmark of pathological autoantibody response [8]. Dysregulated B cells and plasma cells play a pathogenic role in allergic and autoimmune diseases in the upper airway. B cells targeting autoantigens can mediate highly destructive inflammation in the upper respiratory tract. For example, the above-mentioned antineutrophil cytoplasmic antibodies are responsible for granulomatous polyvasculitis, which presents marked symptoms of rhinosinusitis, nasal polyps, rhinitis, and lung inflammation. It is indisputable that dysfunctional immune cells and their products play an undisputed role in nasal mucosa diseases [7,171].

## 6. Genetic Aspects of Nasal Polyp in Autoimmune Responses

Both genetic and environmental factors contribute to the development of autoimmune diseases. The initial immune response to external triggers, such as viral pathogens, generates autoantibodies through molecular mimicry, epitope spreading, and other mechanisms. Meanwhile, genetically susceptible individuals perpetuate the immune response to autoantigens. Support for the potential genetic contribution in CRSwNP stems from observations of familial genetic patterns, with heritability ranging from 14% to 42% in nasal polyps (Table 3) [172]. Cohen et al. divided CRSwNP patients into three categories: nasal polyps, nasal polyps with asthma, and Aspirin-exacerbated respiratory disease (AERD), and found that AERD had the highest heritability (42%), followed by nasal polyps with asthma (30%) and common nasal polyps (15%) [173]. Moreover, the number of family members associated with nasal polyps was correlated with the severity of CRSwNP [173].

Single-nucleotide polymorphisms (SNPs) are single nucleotide variations in DNA sequences that can cause mutations in amino acid sequences when they appear in coding regions of DNA. However, there are many limitations in associating SNPs with nasal polyps in autoimmune pathogenesis. Most genetic studies of nasal polyps only compared SNPs of genes previously implicated in the pathogenesis of nasal polyps, without extensive screening. However, one study found that HLA-DRA polymorphism (P =0.0005–0.02) may play a key role in the development of nasal polyps [174]. Another study found that individuals with HLA-DR7-DQA1*0201 and HLA-DQB1*0202 haplotypes were 2 to 3 times higher odds ratios (ORs) of developing nasal polyps [175]. Fajardo-Dolci et al. reported the HLA-DQA1*0201-DQB1*0201 haplotype was associated with nasal polyp susceptibility and increased the risk of the disease by 5.53 times [176]. Some of these HLA polymorphisms have also been implicated in other autoimmune diseases [177]. Previous studies identified two SNPs in TLR2, Rs3804099, and RS3804100, which showed significant differences between CRSwNP patients and control groups [178]. The gene polymorphism of TLR2 has been investigated in autoimmune diseases such as psoriasis vulgaris and type I diabetes. It is believed that RS3804099 polymorphism of the TLR2 gene is associated with susceptibility to psoriasis vulgaris in southern China [179]. Additionally, rs3804099 is associated with autoimmune diseases in the Chinese Han population [180]. Toll-like receptor 4 (TLR4) and its co-receptor CD14 play a crucial role in autoimmunity by recognizing endogenous ligands, mainly autoantigens. TLR4 and CD14 SNPs may qualitatively and/or quantitatively alter the expression, affecting the susceptibility and severity of systemic lupus erythematosus (SLE) and rheumatoid arthritis (RA) [181]. Nasal polyps are significantly associated with the polymorphism C-159T in the *CD14* gene, which has also been reported in AR and asthma [172,182].

There is increasing evidence suggesting that epigenetic modifications, such as DNA methylation, histone modifications, and gene silencing caused by non-coding RNA, play an important role in the development of autoimmune diseases. For instance, short-chain fatty acids (SCFAs) produced by intestinal microorganisms have been found to promote the differentiation of naive T cells into Treg cells, thereby regulating Treg/Th17 balance and affecting the secretion of IL-17, a pro-inflammatory cytokine involved in autoimmunity, by inhibiting histone deacetylases (HDACs) [45]. The IL-8 promoter is hypomethylated in CRSwNP, which is related to tissue IL-8 protein expression and granulocyte activation [183]. In nasal polyps, hypermethylation of the proximal promoter of the tissue-type plasminogen activator gene (PLAT) can lead to decreased PLAT expression due to negative regulation by Th2 cytokines, resulting in excessive fibrin deposition [184,185]. It is speculated that superantigens, such as SEB, may cause excessive methylation, leading to changes in gene expression in the nasal mucosa and resulting in local inflammation and polyp formation, illustrating that the human microbiota and its metabolites can regulate immune cells and cytokines by epigenetic modifications [88].

**Table 3:** Genetically related mechanisms in CRSwNP.

Genetic Related Mechanisms	Description	Ref.
Positive family histories	14% of patients with CRSwNP have positive family histories.	[186]
	25% had 1 or more first-degree relatives with nasal polyps	[173]
	First-degree relatives(1stDRs) of CRSwNP patients demonstrated a 4.1-fold increased risk of carrying the same diagnosis; second-degree relatives (2ndDRs) demonstrated a 3.3-fold increased risk; no increased risk was observed in spouses of CRSwNP patients.	[187]
Heritability	Heritability ranges from 14% to 42%	[172]
	AERD had the highest heritability (42%), followed by nasal polyps with asthma (30%) and common nasal polyps (15%)	[173]
Single nucleotide polymorphisms associated with autoimmune	HLA-DRA polymorphism	[174]
	HLA-DR7-DQA1*0201 and HLA-DQB1*0202	[175,176]
	SNPs in TLR2: Rs3804099 and RS3804100	[178]
	The polymorphism C-159T in the *CD14* gene	[182]
epigenetic modifications	The promoter of IL-8 is hypomethylated	[183]
	Hypermethylation of proximal PLAT	[185]
	Staphylococcus aureus enterotoxin b (SEB) influences the DNA methylation pattern in nasal polyp tissue	[88]

## 7. Discussion

Recognizing the roles of inflammation, microorganism, and genetics in the autoimmune reaction can provide a clearer understanding of the current autoimmune pathogenesis of nasal polyps. To analyze the occurrence of autoimmune mechanisms in nasal polyps, it is necessary to establish the relationship between nasal polyps and autoimmune reactions from several facts currently studied. Firstly, autoantigen microarrays and other means have identified a variety of autoantibodies that play a role in the occurrence and development of nasal polyps [6,13]. Secondly, the incidence rate of nasal polyps in European and American populations is 1.1% and 2.1–4.4%, respectively [188]. The heritability of patients with nasal polyps ranges from 14% to 42%, highlighting the importance of genetics [172]. Thirdly, there are tertiary lymphoid organs, activated plasma cells, B cells, and disordered Treg cells in nasal polyps [7,107]. These features lead to immunological disorders, autoimmune occurrences, and chronic inflammation of the nasal cavity. Fourthly, *Staphylococcus aureus* is closely related to the occurrence of autoimmune diseases. As mentioned above, *Staphylococcus aureus* promotes autoimmunity through microbial translocation [68], up-regulating MMP to promote the overproduction of autoantigen [49], inducing the formation of NET, acting as superantigens [63] and complementary autoantigens [72], and activating bystander B cells [66], activating immune cells and other ways. Therefore, *Staphylococcus aureus* may also induce or intensify polyp formation through these autoimmune mechanisms.

This paper summarizes many conclusions supporting the important role of autoimmunity in the occurrence of nasal polyps. However, there is currently no clear and widely recognized pathogenic autoantigen identified. No studies have shown that there are autoantibodies and autoreactive T lymphocytes in the animal model of nasal polyps. Nevertheless, we cannot exclude the autoimmune nature of the occurrence and development of nasal polyps. The occurrence of nasal polyps is closely related to the local microenvironment of the nasal cavity, the host’s immune system, and the genetic predisposition. There is increasing evidence suggesting that changes in the composition and function of the nasal microflora can regulate the development and function of the immune system through microbial-dependent mechanisms, thereby interfering with immune homeostasis and ultimately influencing the production of local autoantibodies and local inflammation. By exploring the interaction between the human immune system and the local microenvironment in nasal polyp models, along with host-dependent autoimmune mechanisms and genetic abnormalities of CRSwNP patients, we can gain a better understanding of the mechanisms underlying the development of nasal polyps.

## 8. Contribution to the Field Statement

CRSwNP is a heterogeneous disease characterized by local inflammation and benign hyperplastic growth of nasal mucosa, affecting 1–4% of the general population. It can cause significant long-term symptoms such as nasal obstruction, post-nasal drip, loss of smell, and discharge, seriously affecting patients’ quality of life. While it is generally believed that the barrier function of nasal epithelial cells, the host’s imbalanced immune regulatory system, and the invasion and colonization of pathogenic microorganisms promote the development of CRSwNP. However, recent studies suggest that autoimmunity plays a vital role in developing nasal polyps. Specifically, several autoantibodies have been identified in nasal polyp tissue, along with activated B cells, plasma cells, and dysregulated Treg cells. Additionally, there is a high correlation between nasal polyps and pre-existing autoimmune diseases. There is no review of the pathogenesis of nasal polyps from the perspective of autoimmunity. In this paper, we aimed to fill this gap by demonstrating the significant role of autoimmunity in the pathogenesis of nasal polyps from three distinct perspectives: (1) microbe-mediated autoimmune mechanism, which encompasses autoantigens overproduction, microbial translocation, molecular mimicry, superantigens, inhibition receptors related to autoimmunity; (2) immune abnormalities dependent on the host response, which includes bystander activation, dysregulation of TLRs, epitope spreading, autoantigens complementarity, immune cell response; and (3) genetics, which includes heritability, single nucleotide polymorphisms, and epigenetic modifications.

## Figures and Tables

**Table 1:** The specific antibodies detected in CRSwNP and their roles to induce nasal polyps.

Autoantibodies	Mechanism to Induce Nasal Polyp	Ref.
anti-BP180(BPA2/collagen XVII)	affect the adhesion of the stratified epithelia to the basement membrane	[9]
anti-periplakin IgE	inhibit epithelial repair and lead to chronic airway epithelial injury	[14]
anti-phospholipid(APA)	increase the fibrin deposition that forms the matrix of a nasal polyp	[13]
anti-phosphatidylethanolamine (anti-PE)	associate with coagulation-induced pathology	[13]
anti-IL-5	maintain Th2 inflammation	[12]
anti-IL-17	exert its proallergic effect at the level of B cells, regulate Th2-skewed inflammation	[12]
anti-dsDNA	trigger recurrent inflammation.	[6]
anti-desmoglein 3	cause chronic inflammation	[16]
anti-matrigel IgG	activate the classical pathway	[8]
anti-HSP70	induce the failure of mucosal immunologic tolerance to HSP70 and develop a mucosal autoimmune response	[17]

**Table 2:** Summary of the changes in the microbiota associated with CRS.

Microbiota Change	Description	Ref.
Reduced diversity	Reduced microbial diversity in sinuses of patients with CRS	[20,21]
Increased abundance of certain bacterial species	Increased abundance of specific bacterial species in CRS patients, including *Staphylococcus aureus*, *Streptococcus pneumoniae*, *Haemophilus influenzae*, and various anaerobic species	[23,25,26]
Decreased abundance of certain bacterial species	Decreased abundance of specific bacterial species in CRS patients, including *Bifidobacterium longum, Acinetobacter johnsonii, Corynebacterium, Lactobacillus.*	[23,25,26]
Biofilm formation	Bacterial biofilms in the sinuses of CRS patients can make the bacteria more resistant to antibiotics	[31,32]
Fungal overgrowth	Increased abundance of fungal species in CRS patients, particularly *Aspergillus fumigatu*	[33]
Dysbiosis	Expansion of pathogenic bacteria and reduction of symbiotic bacterial populations	[22]

## Data Availability

Not applicable.
